# Carry‐over effects on the annual cycle of a migratory seabird: an experimental study

**DOI:** 10.1111/1365-2656.12580

**Published:** 2016-08-31

**Authors:** Annette L. Fayet, Robin Freeman, Akiko Shoji, Holly L. Kirk, Oliver Padget, Chris M. Perrins, Tim Guilford

**Affiliations:** ^1^ Oxford Navigation GroupDepartment of ZoologyUniversity of OxfordOxfordUK; ^2^ Institute of ZoologyZoological Society of LondonLondonUK; ^3^ Edward Grey InstituteDepartment of ZoologyUniversity of OxfordOxfordUK

**Keywords:** cost of reproduction, cross‐fostering, etho‐informatics, geolocation, life‐history theory, migration, phenology

## Abstract

1. Long‐lived migratory animals must balance the cost of current reproduction with their own condition ahead of a challenging migration and future reproduction. In these species, carry‐over effects, which occur when events in one season affect the outcome of the subsequent season, may be particularly exacerbated. However, how carry‐over effects influence future breeding outcomes and whether (and how) they also affect behaviour during migration and wintering is unclear.
2. Here we investigate carry‐over effects induced by a controlled, bidirectional manipulation of the duration of reproductive effort on the migratory, wintering and subsequent breeding behaviour of a long‐lived migratory seabird, the Manx shearwater *Puffinus puffinus*. By cross‐fostering chicks of different age between nests, we successfully prolonged or shortened by ∼25% the chick‐rearing period of 42 breeding pairs. We tracked the adults with geolocators over the subsequent year and combined migration route data with at‐sea activity budgets obtained from high‐resolution saltwater‐immersion data. Migratory behaviour was also recorded during non‐experimental years (the year before and/or two years after manipulation) for a subset of birds, allowing comparison between experimental and non‐experimental years within treatment groups.
3. All birds cared for chicks until normal fledging age, resulting in birds with a longer breeding period delaying their departure on migration; however, birds that finished breeding earlier did not start migrating earlier. Increased reproductive effort resulted in less time spent at the wintering grounds, a reduction in time spent resting daily and a delayed start of breeding with lighter eggs and chicks and lower breeding success the following breeding season. Conversely, reduced reproductive effort resulted in more time resting and less time foraging during the winter, but a similar breeding phenology and success compared with control birds the following year, suggesting that ‘positive’ carry‐over effects may also occur but perhaps have a less long‐lasting impact than those incurred from increased reproductive effort.
4. Our results shed light on how carry‐over effects can develop and modify an adult animal's behaviour year‐round and reveal how a complex interaction between current and future reproductive fitness, individual condition and external constraints can influence life‐history decisions.

## Introduction

Animals must balance the cost of current reproduction against survival and the cost of future reproduction (Williams 1966). This is especially true in long‐lived species which can breed for many years and only raise a small number of offspring at each attempt; this makes the adult's own survival and future ability to reproduce a priority over the success of the current brood. When events affecting an adult in one season alter the outcome of the subsequent season, they are generally known in ecology as carry‐over effects (although the concept can be broadened to other life stages and time‐scales: O'Connor *et al*. 2014). Such effects have been demonstrated in a number of taxa including birds and mammals, but evidence suggests that they may also exist in fish, reptiles and invertebrates (Harrison *et al*. 2011).

Carry‐over effects may be exacerbated in migratory species, where survival until the next breeding season relies on undertaking a physically demanding and potentially dangerous return migration to more productive wintering grounds (Alerstam 1990; Norris & Taylor 2006). In adults of these species, a poor non‐breeding season may have important impacts on the next reproductive event, resulting, for example, in more costly migration, reduced body condition the next season or even death (Sillett, Holmes & Sherry 2000; Norris *et al*. 2004; Saino *et al*. 2004a,b). Similarly, the timing of return migration to the breeding site may influence breeding success; many species match their breeding dates to food availability (Regular *et al*. 2014; Hinks *et al*. 2015), and early return often correlates with higher success (Kokko 1999; Bêty, Gauthier & Giroux 2003). Conversely, increased breeding effort may also influence the migratory journey and force animals to migrate with poorer body condition (Marra, Hobson & Holmes 1998), perhaps in turn reducing overwinter survival or condition the next breeding season. Migration phenology may also be affected: a delayed departure on migration due to late breeding could result in more difficult or dangerous migratory conditions (Richardson 1990; Owen & Black 1991). The timing of departure on migration has been shown to depend on the timing of the end of breeding in species such as black‐legged kittiwakes *Rissa tridactyla* (Bogdanova *et al*. 2011), savanna sparrows *Passerculus sandwichensis* (Mitchell *et al*. 2012) and Cory's shearwaters *Calonectris diomedea* (Catry *et al*. 2013), but not in Barnacle geese *Branta* spp. (Jonker *et al*. 2011).

In altricial species, feeding young is costly, and parental effort is mediated by parental response to offspring demand which may lead to parent–offspring conflict (Trivers 1974). Parent–offspring conflict can be greater earlier in the breeding season when parental care is small (Coleman, Gross & Sargent 1985). However, in migratory species this conflict may also be exacerbated towards the end of the breeding period, when it becomes critical for parents to depart on, or prepare for, migration, and a delay could have negative consequences for future reproduction (Alerstam & Lindström 1990). Prioritizing offspring care over favourable migratory timing is therefore likely to have important consequences and could generate carry‐over effects, and so animals may have to make important life‐history decisions about whether to extend care and migrate late, or abandon their offspring. Studies investigating the effect of breeding phenology on the timing of migration and the persistence of carry‐over effects over the annual cycle in migratory species are scarce, mainly due to the challenge of tracking migrants during the non‐breeding season. Often studies stop at the end of breeding (Johnsen, Erikstad & Sæther 1994; Jonker *et al*. 2011; Riou, Chastel & Hamer 2012). Although several studies investigate future survival and reproductive success in response to current breeding effort (Catry *et al*. 2013; Shoji *et al*. 2015), few have manipulated reproductive effort in a controlled way (Johnsen, Erikstad & Sæther 1994; Erikstad *et al*. 2009; Riou, Chastel & Hamer 2012) and investigated potential carry‐over effects on non‐breeding behaviour (Daunt *et al*. 2014; Shoji *et al*. 2015) or more subtle effects on the next breeding season than success or failure (e.g. lay date or offspring quality). Such individual carry‐over effects can influence population dynamics, especially in migratory species (Norris & Taylor 2006); it is therefore important to answer these questions.

To investigate both immediate and delayed (carried over) responses to altered reproductive effort in a long‐distance migrant, and whether and how they influence life‐history decisions, we manipulated the duration of chick‐rearing by cross‐fostering different aged chicks between 42 pairs of breeding Manx shearwaters (*Puffinus puffinus)*. Manx shearwaters are small (c. 400 g), pelagic seabirds breeding primarily in dense colonies in the north‐east Atlantic but overwintering on the Patagonian shelf off South America (Guilford *et al*. 2009), which only raise a single offspring each year. An additional 20 control pairs received a foster chick of the same age as their own. We investigated the carry‐over effects on the subsequent non‐breeding and breeding seasons by using a combination of three types of data. When possible, to separate carry‐over effects from individual quality, we included longitudinal comparisons within treatment or even within individual to compare carry‐over effects within groups between experimental and non‐experimental years. First, we used at‐colony measurements during the experimental breeding season and the next to measure parental effort (daily chick mass gain), breeding phenology (lay date and fledging date) and reproductive performance (chick peak weight and breeding success). Secondly, we recorded the non‐breeding movements with miniature geolocators of all adults following manipulation to measure the timing of autumn and spring migration, wintering and return to the colony. A subset of birds was also tracked with geolocators during the previous and/or the following non‐breeding seasons to allow for within‐group comparisons. Thirdly, we used an etho‐informatics approach to identify different behaviours and estimate year‐round at‐sea activity budgets from saltwater‐immersion data collected by geolocators (Freeman *et al*. 2013), as well as provisioning frequency during the chick‐rearing period.

## Materials and methods

The study was carried out on Skomer Island, Wales (51°44′N, 5°19′W), probably the largest Manx shearwater colony in the world (~300 000 breeding pairs; Perrins *et al*. 2012), between June 2012 and August 2014. All work was conducted after approval by the British Trust for Ornithology Unconventional Methods Technical Panel (permit C/5311), Natural Resources Wales, Skomer Island Advisory Committee and the University of Oxford's Local Ethical Review Process. Bird handling was kept to a minimum: deploying and retrieval of geolocators did not exceed 10 min, and weighing of chicks was <1 min in most cases.

### Cross‐fostering

Study burrows were monitored from the start of the breeding season (late April) to measure laying and hatching dates. This selection of burrows included a subset of burrows for which we had previously tracked birds, burrows from untracked birds we had monitored in previous years and newly monitored burrows (all randomly chosen among a larger sample of each three categories). All breeding adults were ringed with a unique metal ring from the British Trust of Ornithology. Once hatching date was known, nests were allocated to experimental groups (control, ‘longer effort’ treatment or ‘shorter effort’ treatment): control chicks were swapped with chicks of the same age (age difference 0–1 days, *n *=* *20), while chicks in the treatment groups were swapped with chicks 18 days (17·7 ± 0·3 days on average) younger (‘longer effort’ group, *n *=* *21) or older (‘shorter effort’ group, *n *=* *21). This age difference was the largest difference possible given the natural spread of hatching in the study burrows (lay dates usually span over ~40 days from late April to early June, but the majority of laying occurs during a ~15‐day period in mid‐May) and represents c. 30% of the whole chick‐rearing period, which (unlike laying date) is highly consistent among birds and years (Brooke 1990). Therefore, while all laying dates of manipulated nests fell within the natural range, the duration of the shortened or lengthened provisioning period did not. The allocation of nests to a control or treatment group was done as randomly as possible: a burrow could be paired either with another burrow with the same hatching date (control group) or with a burrow with a hatching date 18 days earlier or later (treatment groups). If several burrows matched either or both of these cases, the burrow would be matched randomly with one of them, but if a single burrow matched, it would be paired with this burrow. The process continued until 42 burrows had been allocated to a treatment group (21 ‘longer effort’ and 21 ‘shorter effort’) and 20 burrows to a control group (over the course of 2 years). In total, 62 chicks were cross‐fostered: 30 in 2012 and 32 in 2013 (2012: 10 controls, 10 ‘shorter effort’, 10 ‘longer effort’; 2013: 10 control, 11 ‘shorter effort’, 11 ‘longer effort’). Adults can brood guard young chicks for 5–7 days after hatching. To avoid disturbing chicks during this vulnerable period and to ensure cross‐fostering occurred when parents were away from the nest, we cross‐fostered control chicks and ‘longer effort’ chicks at day 10 and ‘shorter effort’ chicks at day 28. All parents continued feeding their foster chick after cross‐fostering: there was no desertion. Two chicks were missing and one found dead for unknown reasons, respectively, 10, 33 and 7 days after cross‐fostering, despite having been fed regularly by their foster parents. The rate of natural chick death and disappearance (perhaps from gull predation) was similar to, or lower than, that of unmanipulated burrows (Perrins *et al*. 2013). Chicks were weighed daily, during daylight while parents were at sea, to monitor food delivery mass and chick growth until fledging.

### Deployment and retrieval of geolocators

Geolocators (BAS Mk15 and M19, Biotrack M4083), miniature archival light‐loggers (<2·5 g) which also measure the proportion of time immersed in saltwater for every 10‐min bin, were deployed on all adults during chick‐rearing. Most were deployed before or shortly after cross‐fostering to record at‐sea behaviour and nest visit rate. Devices were attached with lightweight cable ties to a tarsus‐mounted plastic ring (for details see Guilford *et al*. 2009), and downloaded when birds were re‐caught back at the colony the following year. Of the 120 devices deployed during experimental years, 27 were not recovered, five failed to collect data and a proportion stopped collecting data before the end of the deployment period. In total, 86 autumn migrations, 78 wintering periods, 70 spring migrations and 85 chick‐rearing periods were recorded fully. In addition, devices were deployed during non‐experimental years (before or after the cross‐fostering event) on a subset of birds, 27 of which collected data (see details in Table S1, Supporting Information).

### Phenology of migration

Twice‐daily positions recorded by geolocators were averaged to obtain daily positions during the non‐breeding season, and erroneous positions were filtered out based on latitude and longitude standard errors and speed (see Supporting information). Migration coincided with equinox periods when the resolution of latitude data is poor, so we used longitude (crossing of the −12° meridian) to determine the start of autumn and the end of spring migrations. This particular longitudinal threshold was chosen to rule out long foraging trips to the Atlantic during the breeding season (Dean *et al*. 2015). The end of autumn and start of spring migrations were calculated as the days on which birds entered or left a 500‐km circle around their average wintering position between November and January.

### Identification of at‐sea behaviour from saltwater‐immersion data

Activity budgets were derived from saltwater‐immersion data collected by geolocators, which record the proportion of time spent submersed in any given 10‐minute period throughout the year. For each bird, the data were split into ‘chick‐rearing’, ‘autumn migration’, ‘wintering’ and ‘spring migration’, using the dates calculated from position data (pre‐breeding and incubation data were not included in the analysis). Hidden Markov models (HMMs) were used to identify and classify behaviours as in Dean *et al*. (2012). These models, which were run on the raw activity data and the daily proportion of time spent dry, use machine‐learning techniques to recognize patterns in the data: given a number of states *K*, they can identify the characteristics of these *K* states, calculate the probability of each data point being generated by each state and classify all data points by their most probably state, while also accounting for spatial autocorrelation between data points. Behaviours were identified and classified for the breeding and wintering periods separately, as different behaviours may be expected to take place during different parts of the birds’ annual cycle. In both periods, three main states were identified, most likely associated with sitting on the water surface, sustained flight and foraging (see Supporting information for details).

### Statistics

To avoid the potentially confounding effects of between‐year variation when testing for differences in breeding phenology, each laying date was formulated as its difference to the median laying date at the colony that year (obtained from a sample of ~100 nests). Analyses of chick manipulation effects on breeding and non‐breeding variables (e.g. lay date, start of migration) used linear mixed models (LMMs) and included year and bird identity (for models run on individual variables, i.e. all non‐breeding variables such as migration phenology or wintering behaviour, and individual foraging effort during breeding) or pair/nest identity (for models run on variables common to a pair, i.e. all variables measured at the nest during the breeding season) as random effects. Previous reproductive success was also controlled for when within‐group differences in winter activity budgets between experimental and non‐experimental years were examined. Dependent variables were transformed to meet normality assumptions when necessary. Paired Wilcoxon and *t*‐tests were used to compare between‐year consistency in laying date and egg mass between groups, and chi‐square tests were used to test for differences in breeding success between groups. LMM *P*‐values were obtained by comparing our models to null models (with random effects but without the fixed effect of interest) with a chi‐square test. All LMMs were performed using the lme4 package in r 3.2.4 (R Core Development Team 2016), and conditional *R* ^2^ values were obtained with the mumin package. Some geolocators stopped collecting data before the end of the non‐breeding season. Incomplete tracks were still included in the analysis, except when calculating variables requiring a complete track (e.g. total time spent foraging in winter). Thus, sample sizes vary slightly between models testing different dependent variables (see Supporting information for details). Furthermore, when analysing breeding variables the year after manipulation, only nests where both manipulated partners returned and bred were included. Nests where a manipulated bird returned to breed with a new partner were excluded.

## Results

**Figure 1:**
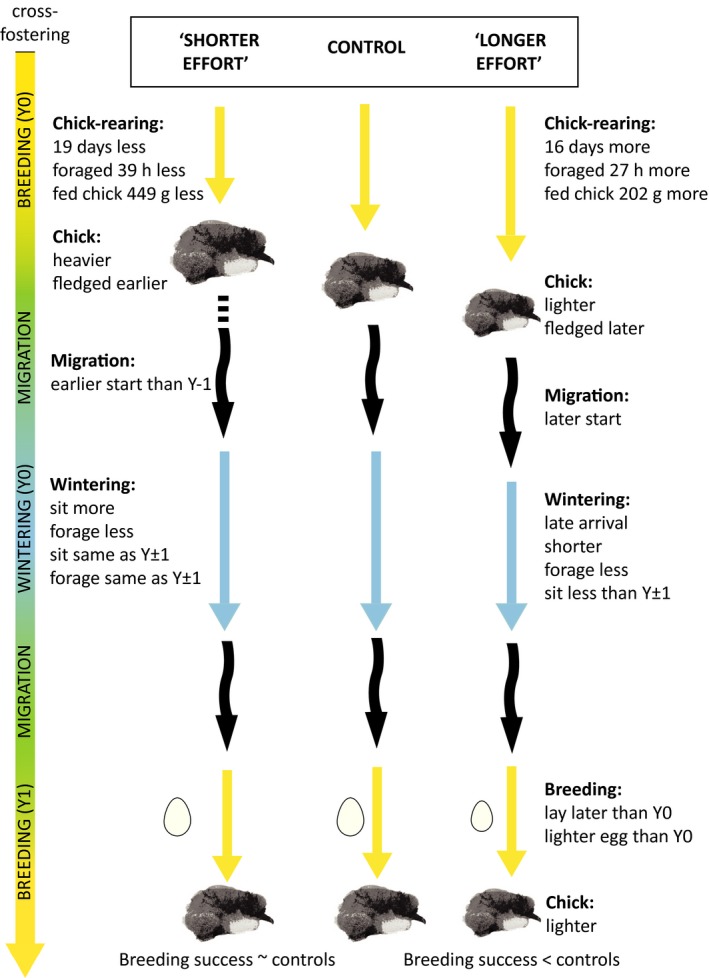
Summary figure of the observed effect of cross‐fostering on the breeding season, following non‐breeding season and subsequent breeding season of all three groups. Only significant differences are indicated (*P *≤* *0·05).

The main results of the study are summarized in Fig. 1. Sample sizes and post hoc test statistics for the second, third, and fifth subsections, unless indicated in the main text, can be found in Table 1.

**Table 1:** Differences between treatment and control groups during the year after cross‐fostering. The ‘control’ column shows the actual values of the control group, and ‘shorter effort’ and ‘longer effort’ columns report the difference between treatments and controls. The test statistics shown are from post hoc analyses, if significant differences were found in a first LMM run on all groups (statistics of which are in the main text). Significant differences (*P* < 0·05) are in bold. Stars next to variables’ names indicate within‐group or within‐individual comparisons between years

	Shorter effort (difference from control)	Control	Longer effort (difference from control)
**Breeding season (year 0)**			
Chick‐rearing duration	**18·9 ± 1·3 days shorter** *n *=* *14, *R* ^2 ^= 0·88, $\chi_{1}^{2}$ = 64·1, *P *<* *0·001	69·4 ± 0·5 days (*n *=* *17)	**16·2 ± 0·9 days longer** *n = *20, *R* ^2 ^= 0·93, $\chi_{1}^{2}$ = 85·7, *P *<* *0·001
Adult foraging time	**40·1 ± 11·9 fewer hours** *n = *19, *R* ^2 ^= 0·72, $\chi_{2}^{2}$ = 15·7, *P *<* *0·001	20·1 ± 4·1 h (*n = *18)	**26·9 ± 16·1 extra hours** *n = *10, *R* ^2 ^= 0·23, $\chi_{2}^{2}$ = 7·4, *P *=* *0·007
Total food given to chick	**449·4 ± 38·9 g less** *n = *19, *R* ^2 ^= 0·78, $\chi_{1}^{2}$ = 59·6, *P *<* *0·001	1077·7 ± 29·7 g (*n = *20)	**207·6 ± 38·6 g more** *n = *20, *R* ^2 ^= 0·64, $\chi_{1}^{2}$ = 20·9, *P *<* *0·001
Chick peak weight	9·2 ± 12·8 lighter (ns) *n = *19, *R* ^2 ^= 0·41, $\chi_{1}^{2}$ = 0·5, *P *=* *0·483	576 ± 12 g (*n = *20)	**39·4 ± 18·8 g lighter** *n = *20, *R* ^2 ^= 0·46, $\chi_{1}^{2}$ = 4·3, *P *=* *0·038
Chick fledging weight	**37·1 ± 12·9 g heavier** *n = *19, *R* ^2 ^= 0·52, $\chi_{1}^{2}$ = 6·7, *P *=* *0·009	418 ± 10 g (*n = *20)	**51·7 ± 19·2 g lighter** *n = *20, *R* ^2 ^= 0·45, $\chi_{1}^{2}$ = 6·7, *P *=* *0·009
Chick fledging date	**5·8 ± 1·8 days earlier** *n = *19, *R* ^2 ^= 0·22, $\chi_{1}^{2}$ = 10·1, *P *=* *0·001	6 Sept. ± 0·8 day (*n = *20)	**12·5 ± 1·5 days later** *n = *20, *R* ^2 ^= 0·73, $\chi_{1}^{2}$ = 36·9, *P *<* *0·001
**Non‐breeding season**			
Start of autumn migration	1·3 ± 2·1 days earlier (ns) *n = *23, *R* ^2 ^= 0·33, $\chi_{1}^{2}$ = 0·4, *P *=* *0·556	15 Sept. ± 1·3 days (*n = *30)	**5·3 ± 2·2 days later** *n = *37, *R* ^2 ^= 0·27, $\chi_{1}^{2}$ = 6·0, *P *=* *0·014
Comparison with year − 1*	**8·2 ± 4·1 days earlier than year − 1** *n = *35, *R* ^2 ^= 0·34, $\chi_{1}^{2}$ = 4·1, *P *=* *0·042	5·3 ± 5·5 days later than year − 1 (ns) *n = *35, *R* ^2 ^= 0·43, $\chi_{1}^{2}$ = 1·03, *P *=* *0·310	**9·5 ± 3·9 days later than year − 1** *n = *47, *R* ^2 ^= 0·26, $\chi_{1}^{2}$ = 4·7, *P *=* *0·029
Arrival at the wintering grounds	**0·6 ± 0·4 days later** *n = *23, *R* ^2 ^= 0·18, $\chi_{1}^{2}$ = 3·7, *P *=* *0·047	13 Oct ± 1·6 days (*n = *28)	**1·5 ± 0·4 days later** *n = *35, *R* ^2 ^= 0·38, $\chi_{1}^{2}$ = 9·2, *P *<* *0·001
Overwintering duration	−3·3 ± 4·5 days shorter (ns) *n = *18, *R* ^2 ^= 0·10, $\chi_{1}^{2}$ = 0·5, *P *=* *0·481	142·5 ± 2·2 days (*n = *28)	**11·9 ± 3·2 days shorter** *n = *32, *R* ^2 ^= 0·66, $\chi_{1}^{2}$ = 13·3, *P *<* *0·001
Total time away from colony	5·6 ± 3·5 days more away (ns) *n = *17, *R* ^2 ^= 0·15, $\chi_{1}^{2}$ = 2·7, *P *=* *0·101	211·2 ± 2·4 days away (*n = *25)	**6·8 ± 3·0 fewer days away** *n = *28, *R* ^2 ^= 0·09, $\chi_{1}^{2}$ = 5·0, *P *=* *0·025
**Breeding season (year + 1)**			
Laying date* (paired Wilcoxon test)	2·3 ± 1·7 days later in year + 1 (ns) *n *=* *6, *W* = 11·5, *P *=* *0·343	6·7 ± 2·9 days later in year + 1 (ns) *n *=* *10, *W* = 38·5, *P *=* *0·070	**10·3 ± 3·2 days later in year + 1** *n *=* *10, *W* = 53·5, *P *=* *0·009
Egg mass* (paired *t*‐test)	3·4 ± 3·9 g heavier in year + 1 (ns) *n *=* *5, *t*_4 _= 0·86, *P *=* *0·440	0·1 ± 1·2 g lighter in year + 1 (ns) *n *=* *10, *t*_9 _= −0·09, *P *=* *0·933	**3·2 ± 1·1 g lighter in year + 1** *n *=* *10, *t*_9 _= −2·84, *P *=* *0·019
Chick peak weight	40·3 ± 30·9 g lighter (ns) *n *=* *4, *R* ^2 ^= 0·19, $\chi_{1}^{2}$ = 2·0, *P *=* *0·159	586·8 ± 22·9 g *(n *=* *5)	**51·5 ± 24·9 g lighter** *n *=* *5, *R* ^2 ^= 0·82, $\chi_{1}^{2}$ = 3·9, *P *=* *0·046
Breeding success (chi‐square test)	12·4% lower (ns) *n *=* *21, $\chi_{1}^{2}$ = 0·16, *P *=* *0·691	73·9% (*n *=* *28)	**36·9% lower** *n *=* *36, $\chi_{1}^{2}$ = 5·4, *P *=* *0·020

### Natural differences in breeding phenology before manipulation

Because of the design of the experiment, ‘longer effort’ nests, which had to hatch a chick 18 days before their paired ‘shorter effort’ nest, tended to be early breeders. Conversely, ‘shorter effort’ nests tended to be late breeders, while most control nests, having to be matched with a nest hatching on the same day, tended to breed during the peak of breeding at the colony. Therefore, prior to cross‐fostering ‘shorter effort’ birds laid later than control birds (11·3 ± 1·9 days later on average, LMM: *n*_control _= 12, *n*_shorter_effort _= 12; *R* ^2 ^= 0·63, $\chi_{1}^{2}$ = 23·4, *P *<* *0·001), and ‘longer effort’ birds tended to lay slightly earlier (3·2 ± 1·6 days earlier on average, LMM: *n*_longer_effort _= 14; *R* ^2 ^= 0·19, $\chi_{1}^{2}$ = 3·5, *P *=* *0·060). The pattern of laying date by the same individuals was similar the previous year (Supporting information), supporting findings from previous studies that lay date is highly repeatable within individuals between years (Brooke 1978, 1990). Additional analyses of individual consistency in laying date and egg mass of 44 unmanipulated pairs during the 2011–2014 period also supported these claims (Supporting information).

### Effects of cross‐fostering on current breeding

Cross‐fostering induced differences among groups during the manipulated season (Table 1). Chick‐rearing duration differed between groups (LMM: *R* ^2 ^= 0·94, $\chi_{2}^{2}$ = 146·0, *P *<* *0·001). Adults which raised an older foster chick had a shorter chick‐rearing period (from hatching to fledging: 50·5 ± 1·4 days) than controls (69·4 ± 0·5 days), which spent less time rearing a chick than adults with a younger foster chick (85·6 ± 0·7 days). Manipulation shortened chick‐rearing by 27·2% on average in the ‘shorter effort’ treatment and lengthened it by 23·3% on average in the ‘longer effort’ treatment.

Using the saltwater‐immersion data to calculate visit rate and at‐sea activity budgets during the chick‐rearing period immediately following the manipulation, we found no differences in activity budgets among treatments. All groups spent similar daily amounts of time in sustained flight, foraging or sitting on the water surface (*n*_control _= 24, *n*_shorter_effort _= 23, *n*_longer_effort _= 37, sitting: LMM, *R* ^2 ^= 0·14, $\chi_{2}^{2}$ = 1·1, *P *=* *0·566; foraging: LMM, *R* ^2 ^= 0·13, $\chi_{2}^{2}$ = 0·62, *P *=* *0·732; flying: LMM, *R* ^2 ^= 0·09, $\chi_{2}^{2}$ = 0·6, *P *=* *0·742). Similarly, nest visit rate did not differ between adults of different groups (control: 0·67 ± 0·02 visits day^−1^, *n *=* *25; ‘shorter effort’: 0·68 ± 0·03 visits day^−1^, *n *=* *23; ‘longer effort’: 0·67 ± 0·02 visits day^−1^, *n *=* *37; LMM, *R* ^2 ^= 0·02, $\chi_{2}^{2}$ = 0·05, *P *=* *0·973). However, accumulated time spent in a foraging state (from the 30th day of chick‐rearing until the final feeding event) varied between groups (LMM, *R* ^2 ^= 0·75, $\chi_{2}^{2}$ = 32·2, *P *<* *0·001), with birds in the ‘shorter effort’ treatment spending less time foraging than controls (20·1 ± 4·1 h vs. 59·3 ± 10·9 h) and birds in the ‘longer effort’ treatment spending more (86·3 ± 7·2 h). As a result, total food mass fed to the chicks also varied between groups (LMM: *R* ^2 ^= 0·87, $\chi_{2}^{2}$ = 108·9, *P *<* *0·001), with pairs in the ‘shorter effort’ treatment delivering less (628·4 ± 25·2 g) than controls (1077·7 ± 29·7 g) and ‘longer effort’ treatment pairs delivering more (1279·6 ± 26·1 g).

Fledging age (68·2 ± 0·5 days) was unaffected by treatment (LMM: *n*_control _= 19, *n*_shorter_effort _= 20, *n*_longer_effort _= 20; *R* ^2 ^= 0·02, $\chi_{2}^{2}$ = 1·3, *P *=* *0·530). However, there were differences between the peak weight, fledging weight and fledging date of chicks in different groups (LMMs: peak weight: *R* ^2 ^= 0·46, $\chi_{2}^{2}$ = 6·1, *P *=* *0·048; fledging weight: *R* ^2 ^= 0·53, $\chi_{2}^{2}$ = 19·9, *P *<* *0·001; fledging date: *R* ^2 ^= 0·65, $\chi_{2}^{2}$ = 63·7, *P *<* *0·001, Table 1). Chicks in ‘shorter effort’ nests reached a similar peak weight to controls (569 ±7 g vs. 576 ± 12 g) but fledged both heavier (453 ± 10 g vs. 418 ± 10 g) and earlier in the season (5·8 ± 1·8 days earlier on average). Chicks in ‘longer effort’ nests, on the other hand, reached lower peak weights than controls (538 ± 15 g), and fledged both lighter (367 ± 18 g) and later (12·3 ± 1·6 days later on average).

### Effects of cross‐fostering on migration phenology

**Figure 2:**
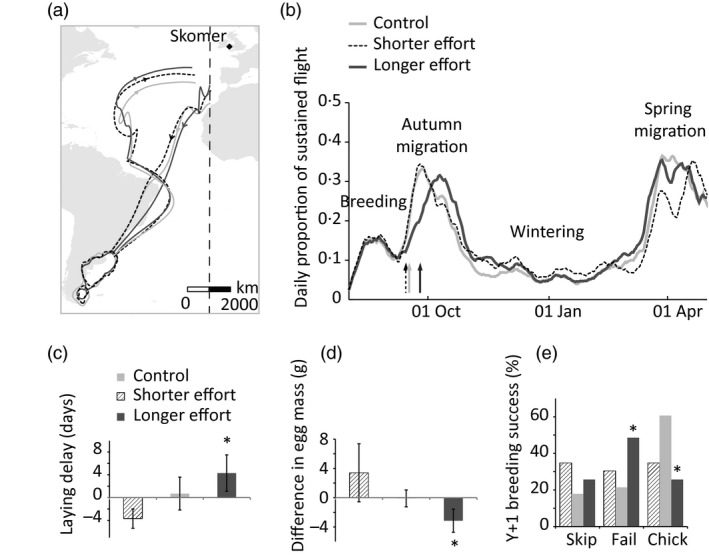
(a) Average autumn and spring migration routes and overwintering areas of control and treatment groups during the non‐breeding season following cross‐fostering (control = light grey, ‘shorter effort’ = dashed black and white, ‘longer effort’ = dark grey). The −12° meridian used to identify the start of autumn (southbound) migration is represented with a vertical line. 95% occupancy kernels for each group during the wintering period are shown (kernels calculated in ArcGIS 10, cell size = 25km, bandwidth = 225km). The breeding colony is indicated with a black diamond. (*b*) Average daily proportion of time in sustained flight for each group, following cross‐fostering. For ease of representation only 2013–2014 is displayed and smoothed over 7 days. Arrows indicate the start of autumn migration (crossing of the −12° longitude) for each group. (*c*) Within‐pair difference in laying date between Y0 (manipulation) and Y+1. (*d*) Within‐pair difference in egg mass between Y0 (manipulation) and Y+1. (*e*) Breeding success one year after manipulation (excluding birds not recaptured). ‘Skip’ refers to birds present on the colony but not breeding, ‘fail’ refers to birds which laid an egg and then failed at egg or chick stage, ‘chick’ refers to birds which successfully fledged a chick. Asterisks indicate significant differences (*: *P *≤* *0·05). Means ± SE.

Treatment did not affect the routes taken during spring and autumn migrations, or the wintering area (Fig. 2a), but did affect the timing of autumn migration (Table 1, Fig. 2b). There was no difference in the start of migration for control birds between their experimental and non‐experimental years, but ‘shorter effort’ birds started migration earlier than in non‐experimental years, while ‘longer effort’ birds started migration later in the experimental year.

Comparisons between groups during the experimental year also showed differences in the timing of autumn migration following manipulation (LMM: *R* ^2 ^= 0·25, $\chi_{2}^{2}$ = 10·8, *P *=* *0·005). Control and ‘shorter effort’ treatments started autumn migration on similar dates but ‘longer effort’ birds left later than controls. Arrival at the wintering grounds was marginally earlier in control than in ‘shorter effort’ birds but ‘longer effort’ birds arrived later than controls.

There was no difference between groups in spring migration departure timing, with ‘shorter effort’ and ‘longer effort’ birds leaving, respectively, 0·5 ± 0·3 days later and 0·2 ± 0·3 days earlier than the average departure date of controls (LMM, *n*_control _= 28, *n*_shorter_effort _= 18, *n*_longer_effort _= 32, *R* ^2 ^= 0·09, $\chi_{2}^{2}$ = 4·46, *P *=* *0·107). Overall, time spent on the wintering grounds differed between groups (LMM, *R* ^2 ^= 0·24, $\chi_{2}^{2}$ = 10·5, *P *=* *0·005). ‘Shorter effort’ and control birds were similar (139·4 ± 4·3 vs. 142·5 ± 2·2 days), but ‘longer effort’ birds spent less time wintering than controls (131·5 ± 1·9 days). Timing of return to the colony did not differ among treatments, with ‘shorter effort’ and ‘lower effort’ birds arriving, respectively, 5·6 ± 3·4 and 0·4 ± 2·7 days later than control birds, on average (LMM, *n*_control _= 17, *n*_shorter_effort _= 25, *n*_longer_effort _= 28, *R* ^2 ^= 0·19, $\chi_{2}^{2}$ = 3·69, *P *=* *0·158). Overall, total time away from the colony differed between treatment and control individuals (LMM, *R* ^2^ = 0·16, $\chi_{2}^{2}$ = 12·78, *P *=* *0·002). On average, ‘longer effort’ birds spent 6·8 ± 3·0 fewer days away than controls, while ‘shorter effort’ birds spent 5·6 ± 3·5 more days away than controls (despite a strong effect size, the latter difference was not statistically significant, perhaps because of the small sample).

### At‐sea behaviour during the wintering following manipulation

We used saltwater‐immersion data to estimate activity budgets during winter (Fig. S1, see Table S2 for details of all statistical tests). ‘Shorter effort’ birds spent more time sitting on the water surface at the wintering grounds than controls (LMM, *n*_control _= 26, *n*_shorter_effort _= 20, sitting: *R* ^2 ^= 0·67, $\chi_{1}^{2}$ = 20·6, *P *<* *0·001). In addition, birds from both treatment groups foraged less than control birds (LMM, ‘shorter effort’: *R* ^2 ^= 0·73, $\chi_{1}^{2}$ = 37·7, *P *<* *0·001; ‘longer effort’: *n *=* *32, *R* ^2 ^= 0·32, $\chi_{1}^{2}$ = 7·13, *P *=* *0·008). The birds’ activity budgets differed during the winter following manipulation compared with other (non‐experimental) winters in terms of foraging and sitting on the surface (LMMs, *n*_control _= 27, *n*_shorter_effort _= 22, *n*_longer_effort _= 35, sitting: *R* ^2 ^= 0·66, $\chi_{2}^{2}$ = 77·24, *P *<* *0·001; foraging: *R* ^2 ^= 0·73, $\chi_{2}^{2}$ = 95·7, *P *<* *0·001) but not flight (LMM, *R* ^2 ^= 0·15, $\chi_{2}^{2}$ = 1·3, *P *=* *0·535). ‘Longer effort’ birds spent less time sitting on the water and tended to forage less in the winter following cross‐fostering than in other years (LMM, sitting: *R* ^2 ^= 0·76, $\chi_{1}^{2}$ = 4·5, *P *=* *0·035; foraging: *R* ^2 ^= 0·77, $\chi_{1}^{2}$ = 3·8, *P *=* *0·051). Conversely, control and ‘shorter effort’ birds spent similar amounts of time sitting and foraging in all years (LMMs, controls: sitting: *R* ^2 ^= 0·42, $\chi_{1}^{2}$ = 1·9, *P *=* *0·172, foraging: *R* ^2 ^= 0·39, $\chi_{1}^{2}$ = 1·6, *P *=* *0·200; ‘shorter effort’: sitting: *R* ^2 ^= 0·44, $\chi_{1}^{2}$ = 0·3, *P *=* *0·612, foraging: *R* ^2 ^= 0·58, $\chi_{1}^{2}$ = 0·6, *P *=* *0·452).

### Effects on the next breeding season

We found differences between treatments in the phenology and outcome of the breeding season one year after manipulation (Table 1, Fig. 2). Control birds laid eggs marginally later and of similar mass (57·5 ± 1·0 g vs. 57·6 ± 1·0 g) as they did the previous year (the year of the manipulation), and their breeding success was similar to that of unmanipulated birds (measured in a neighbouring plot, Perrins *et al*. 2012, 2013). Conversely, ‘longer effort’ treatment birds laid later the year after the manipulation than in the year of the manipulation, and their eggs were lighter than the year before (55·6 ± 1·3 g vs. 58·8 ± 1·0 g). Their chicks reached lower peak weights than chicks from control birds (520·2 ± 34·7 g vs. 586·8 ± 22·9 g). Overall ‘longer effort’ treatment birds also had a lower breeding success (37·0%) than control birds (73·9%). Comparison of ‘longer effort’ pairs to unmanipulated pairs which laid at the same time in the experimental year showed that the shifts observed in laying date and egg mass after manipulation did not occur in unmanipulated birds, and so can be confidently attributed to an effect of the manipulation and not to a return to the pair's average state after unusual early laying the previous year (Supporting information).

Differences were not statistically significant for ‘shorter effort’ treatment birds. They laid at similar dates before and after manipulation, but the delay in laying date compared with controls was reduced from 11·3 ± 1·9 days before manipulation to 3·4 ± 1·7 days the year after. Their eggs were heavier than in the previous year (58·2 ± 1·9 g vs. 54·8 ± 2·9 g), but this difference was not statistically significant. Their chicks reached similar peak weights to controls (546·5 ± 20·8 g) and their breeding success (61·5%) was also similar.

The manipulation did not affect the probability of skipping breeding the year following cross‐fostering, with 13·9%, 21·6% and 21·9% of birds skipping breeding in the control, ‘shorter effort’ and ‘longer effort’ treatments, respectively ($\chi_{2}^{2}$ = 2·6, *P *=* *0·274). Out of 16 pairs which skipped breeding the next year, six did so in their usual pair (i.e. both members were present at the colony but did not breed); in the remaining 10 nests, one of the two birds was not seen on the colony all year.

## Discussion

Cross‐fostering resulted in extension or shortening of the normal chick‐rearing duration by about 25%, which was sufficient to generate differences in reproductive effort and detectable long‐term carry‐over effects. All chicks fledged at a similar age, resulting in adults in manipulated nests expending different levels of effort on their presumed chick: treatment birds finished the chick‐rearing period earlier or later than controls, and there were differences between groups in the total amount of food delivered to the chick. These results show that parents responded to cross‐fostering by adapting their chick‐rearing period to match the demands of the chick (presumably via responsiveness to the chick begging), instead of simply provisioning for a fixed period. Manipulated parents had similar feeding frequency and activity budgets to controls during chick‐rearing, which suggests that they did not alter their daily chick‐rearing behaviour after cross‐fostering, but continued to feed at the same rate until the chick reached an acceptable condition for fledging. While short‐lived altricial species have been found to adjust the duration of care to offspring requirements (Swanson & Campbell 1980; Rehling *et al*. 2012), results are mixed in long‐lived migratory seabirds. Atlantic puffins *Fratercula arctica* and Cory's shearwaters *Calonectris diomedea* with experimentally prolonged parental care adapted their parental effort to chick condition but did not extend their provisioning period (Johnsen, Erikstad & Sæther 1994; Catry *et al*. 2006), while Manx shearwaters adjusted to the new chick age but lowered their provisioning frequency with time, regardless of receiving a younger or older chick (Riou, Chastel & Hamer 2012). As in Riou, Chastel & Hamer (2012), our results support an adaptation to the needs of the foster chick. The difference between their study and ours in terms of feeding frequency may be due to different measures of provisioning rate or to a larger and perhaps more noticeable change in chick age in our study (~18‐day difference vs. ~11 days in Riou, Chastel & Hamer 2012). Contrary to theoretical expectations (Drent & Daan 1980), the ‘longer effort’ birds made the decision to continue provisioning their chick over starting migration on time, potentially lowering their future chances of successful breeding. Perhaps the extension of care was not sufficient to affect the adults’ body condition enough to threaten a successful migration and therefore induce the decision to abandon the offspring. Despite the difference in overall work required to complete provisioning, manipulation did not apparently lead to changes in daily foraging effort at sea when measured by immersion data. However, chicks in the ‘shorter effort’ group fledged heavier than control chicks, while chicks in the ‘longer effort’ group fledged lighter. This may simply be an effect of fledging date: fledging age was similar across groups, and therefore, the older chicks in the ‘shorter effort’ group fledged earlier than control chicks, which fledged earlier than the younger chicks in the ‘longer effort’ group. Fledging date and mass are negatively correlated in Manx shearwaters, with chicks fledging earlier generally being heavier; earlier and heavier chicks also have a higher chance of survival (Perrins 1966). This may be a consequence of a better match between resource availability and chick need, as observed in many species, ith resources being more plentiful earlier in the season (Regular *et al*. 2014; Hinks *et al*. 2015).

We were able to detect the carry‐over effects of the manipulation not only by comparing the treatment groups to the control group but also by making within‐individual and within‐group comparisons between experimental and non‐experimental years. A key result of our study is that manipulating chick‐rearing duration affected not only the timing of migration but also the behaviour of birds during the non‐breeding season. The timing of migration departure has been shown to depend on the end of breeding in several species of seabirds, with failed breeders starting migration earlier than successful ones (Bogdanova *et al*. 2011; Guilford *et al*. 2012; Catry *et al*. 2013), but this is not always so in other migratory birds (Jonker *et al*. 2011). In agreement with other seabird studies, we found that shearwaters with an extended provisioning period started migrating later than they normally do in other years (and later than controls), while birds with a shortened chick‐rearing period started their migration earlier than in other years (but no earlier than controls). Theoretical models suggest that the decision to start migration should depend heavily on body condition and that birds which migrate late after breeding may not recover sufficient reserves in time for the following breeding event (McNamara, Welham & Houston 1998). However, they also show that different selection pressures may apply on spring and autumn migration with timing more critical in spring. Consistent with these predictions all our birds started spring migration at similar times, regardless of when they had reached the wintering grounds. Taken together, our results suggest that the timing of breeding does affect the timing of autumn but not spring migration. Furthermore, there seems to be no benefit of leaving on migration as early as possible (‘shorter effort’ birds did not leave the colony earlier than controls despite finishing breeding earlier), but rather that there is an ideal timing of migration, which may be missed by late breeders. The timing of migration is affected by environmental factors and quality of the wintering grounds in passerines and waders (Marra, Hobson & Holmes 1998; Forchhammer, Post & Stenseth 2002; Hüppop & Hüppop 2003; Gunnarsson *et al*. 2006) and this may also be the case in seabirds.

In addition to affecting the timing of autumn migration, the manipulation also affected behaviour at the wintering grounds. Despite all birds following similar migratory routes and visiting similar wintering grounds, birds differed in their daily activity budgets. Compared with control birds during the same winter, all manipulated birds spent less time engaged in foraging‐related activities. Birds with a shortened provisioning period also spent more time sitting on the water surface. Why this is the case is unclear and may be due to inherent differences in individual quality between groups due to the design of the experiment. Between‐year comparisons within each treatment allowed us to bypass this issue and showed that ‘longer effort’ birds reduced the amount of foraging and time sitting on the water surface daily in the winter following manipulation. This was also accompanied by a not statistically significant increase in flight behaviour (perhaps due to a small sample of birds tracked in non‐experimental years); since the birds were already at the wintering grounds, this flight is likely related to searches for food on greater scales. These changes in behaviour suggest an effort to regain body condition, prompted by a longer breeding season and perhaps also by a shortened wintering period. In contrast, control and ‘shorter effort’ birds did not change their daily behaviour between years. Winter foraging activity has been shown to be a good predictor of the probability of skipping breeding the following season in Manx shearwaters (Shoji *et al*. 2015), and European shags *Phalacrocorax aristotelis* which forage more in winter tend to do less well the following breeding season (Daunt *et al*. 2014). This suggests that birds adapt their winter foraging effort as a constrained response to their body condition rather than as an active decision concerning survival and future breeding. In agreement with Shoji *et al*.'s results, we found that birds allocating more effort into one breeding season had a lower breeding success the next. However, our studies differ on the direction of the correlation between winter foraging activity and subsequent reproductive performance. While we found that ‘longer effort’ birds foraged less overwinter after manipulation than in non‐experimental years, other studies found the opposite: the more the birds foraged in winter, the more likely they were to breed late (Daunt *et al*. 2014) or to skip breeding altogether (Shoji *et al*. 2015). The reason for these differences is unclear, but may be due to different magnitudes of carry‐over effects (those induced by breeding or skipping vs. those induced by a 25% change in chick‐rearing duration), or differences in measurements of foraging activity. We also cannot rule out the possibility that birds in our study recognized that their foster chick was not their own and that this somehow affected their behaviour. This seems unlikely, however, as we did not notice any change in provisioning behaviour after the manipulation, and several species of shearwaters have been shown to provision a foster chick normally (Hamer & Hill 1994).

Carry‐over effects from the manipulation were also visible during the next breeding season, despite similar return dates to the colony among groups – usually a strong predictor of breeding performance in migratory species (Kokko 1999; Bêty, Gauthier & Giroux 2003). Birds that had provisioned a chick for longer in the experimental year had a lower breeding success than control birds, while the breeding success of birds with a reduced parental effort the year before had a similar breeding success to controls. Interestingly, the proportion of birds skipping breeding was similar across groups, and similar to the norm (Brooke 1990). Perhaps the manipulation was too subtle to prompt skipping or the conditions at the wintering grounds were good enough to allow most birds to regain sufficient condition to attempt breeding the next year. However, the number of birds not seen the year following the experiment, which may or may not have been skipping breeding, makes a firm conclusion difficult. Nonetheless, despite ‘longer effort’ birds not making the decision to skip breeding more than other groups, they did have lower breeding success, mostly resulting from eggs failing to hatch. Incubation can be a demanding period in Manx shearwaters, with parents alternating long fasting incubation stints at the nest and adults losing up to 25% of their body weight during a single stint (Harris 1966). This high rate of incubation failure suggests that ‘longer effort’ birds were in a poorer body condition at the start of the following season, but decided to attempt breeding nonetheless. Those who succeeded in rearing a chick also suffered visible carry‐over effects from the manipulation, raising lighter chicks than the previous year. These birds’ decision not to skip breeding (or not to abandon their foster chick the previous year) does not match theoretical predictions (Drent & Daan 1980; Shaw & Levin 2012). Perhaps the carry‐over effects incurred by the extended care the previous year were not sufficient to justify skipping, but the low success rate of those who attempted breeding suggests otherwise. It may be that the ‘longer effort’ birds were naturally higher quality breeders (the design of the experiment favouring them to be early breeders) which do not usually skip breeding. Individual consistency in intermittent breeding has been observed in other species (Cam *et al*. 1998), and could also explain why we also did not observe a lower skipping rate in ‘shorter effort’ birds (which tended to be natural late breeders, so of lower quality).

Lay date, egg size and chick mass are usually good proxies for egg quality, hatching and fledging success and chick survival in several species of birds, including seabirds (Parsons 1970; Perrins, Harris & Britton 1973; Schifferli 1973; Croxall, Rothery & Crisp 1992). Female Manx shearwaters tend to lay eggs of similar size at similar dates each year (Brooke 1978, 1990; and additional analyses in the Supporting information), which led to the claim that, age and pair bond duration aside, external factors are of little influence on laying (Brooke 1990). Our results demonstrate that increased reproductive effort can affect subsequent laying date and egg size. Indeed, we found carry‐over effects of reproductive effort (and most likely of foraging effort the previous winter) on laying date, egg size and chick quality the following year. ‘Longer effort’ birds laid eggs later and smaller than they did the previous year, and their chicks reached lower peak weights. Control birds, on the other hand, laid similar‐sized eggs at a similar date to the year before. Although the differences were not statistically significant, ‘shorter effort’ birds tended to lay earlier and bigger eggs than before. They had similar breeding success and chick peak weight to controls. The lack of significant difference in laying date and egg mass could be due to low statistical power (*n *<* *7 for within‐individual comparisons). Alternatively, ‘shorter effort’ birds may inherently be lower quality breeders because of the experimental design (they laid later than others), and therefore, the benefits from a shortened chick‐rearing duration may have been masked by lower parental quality. Nonetheless, our results suggest that a reduction in reproductive effort may also incur ‘positive’ carry‐over effects to the following non‐breeding and breeding season, but that these effects last less long, or are less strong, than those provoked by an increased reproductive effort. In other words, the carry‐over effects are asymmetric so that at least when reproductive effort is externally manipulated, the future benefits of a current reduction are less marked than the future costs of an equivalent current increase.

Both the differences in total provisioning effort and in timing of cessation of parental care may have contributed to the carry‐over effects observed in our study, but the design of our experiment does not allow us to disentangle these effects. It is also important to note that our results are not simply due to the difference in natural lay date between treatment groups. If this were the case, ‘longer effort’ birds (early, high‐quality breeders) would have done better than ‘shorter effort’ birds (late, poorer quality breeders), the opposite of what we observed. Therefore, the imbalance between treatment groups can only have led to an underestimation of the strength of the effects we observed. Furthermore, the similarity in the between‐group differences in laying dates between the experimental year and the previous year, combined with the results of additional analyses of unmanipulated pairs (Supporting information), shows that laying date is repeatable in non‐experimental conditions. Therefore, the differences in laying dates prior to manipulation were not themselves due to carry‐over effects from earlier seasons, neither were the differences in breeding parameters observed after manipulation a simple return to an average state after an exceptionally good or poor year.

In summary, life‐history theory recognizes that cost‐benefit trade‐offs between growth, survival and reproduction select both for structurally different life styles between species, and potentially for flexibility to respond to fitness‐changing conditions during an individual's life. Carry‐over effects demonstrate, as here during adulthood, the strategic connections between a decision (or lack of it) at one stage and the consequences at another (later) stage in life. As recent theoretical treatments emphasize (O'Connor *et al*. 2014; Senner, Conklin & Piersma 2015), understanding the mechanisms that organisms employ to reverse the changes that drive adverse carry‐over effects, and the time‐scales over which these may be dissipated are emerging priorities in ecological research. These issues underpin the potential responsiveness of individuals, and ultimately populations, to environmental perturbations, and they reflect how the tensions between constraint and strategic response have been resolved by evolution in the organism's life history. In our study, we show that costs borne by an adult individual in one breeding season, as a consequence of experimental intervention, feed forward at least into the following breeding season. We see these as between‐group effects on reproductive success, but also as longitudinal changes within individuals in reproductive variables such as timing of laying, egg size and chick weight. Carry‐over effects can be induced both positively and negatively by manipulating reproductive effort, potentially asymmetrically. Individuals might of course respond differently, as may the two sexes, and these complexities remain to be resolved. The fact that we also observe changes in behaviour during the intervening winter and migrations which are potentially compensatory suggests that affected individuals work to dissipate these carry‐over effects as they continue their lives, making them ‘reversible state effects’ in the terminology of Senner, Conklin & Piersma (2015). As yet, however, we do not know just how long this dissipation may take, or indeed whether some individuals may be pushed into a vicious cycle of late migration and poorer condition by such carry‐over effects (e.g. as observed in black‐tailed godwits: Gunnarsson *et al*. 2005); this could be determined by following manipulated adults over a longer time period. Thus, we verify that the long‐lived life style of species like the Manx shearwater is threaded with the long‐term trade‐offs that underpin life‐history theory, and which help to explain functional behavioural diversity. However, we also show that shearwaters, at least under the conditions of our experiment, appear to persist in the reproductive attempt on which they embarked before manipulation (despite an approximately 25% increase in chick‐rearing effort) even though it will lead them to suffer at least year‐long consequences. This raises the possibility that individually birds do not in fact have much flexibility to respond dynamically to an unexpected change in fortunes during breeding, and instead that breeding decisions which fall short of catastrophic consequences (e.g. personal starvation) are perhaps made in response to conditions early during the attempt. Nevertheless, parents could have decided to stop feeding their foster chick and so, alternatively, it may be that the effort costs we induced here were in fact small relative to the fitness costs of abandoning the chick despite the demonstrated carry‐over effects and that birds are indeed capable of optimising their life‐history decisions dynamically during breeding. Fundamental questions such as this, concerning the time‐scales over which long‐lived organisms optimize life‐history decisions, the degree to which behaviour is periodically canalized or dynamic, remain little understood.

## Data accessibility

Data are available from the Dryad Digital Repository http://dx.doi.org/10.5061/dryad.32kc7 (Fayet *et al*. 2016).

## Supporting information


**Appendix S1.** Supplementary Methods.
**Fig. S1.** Example of the classification in six states of a 24 h‐period during the breeding season.
**Fig. S2.** (a) Example of behavioural classification of saltwater‐immersion data by a HMM for a day in the wintering period of a bird.
**Table S1.** Number of complete tracks collected during each period of the annual cycle (excluding pre‐laying and incubation), for experimental and non‐experimental years.
**Table S2.** Comparison of activity budgets during the wintering period.
[Correction added after online publication on 19 September 2016: Supporting information file replaced, as original was corrupt].
