# Supplementary material for: Carry‐over effects on the annual cycle of a migratory seabird: an experimental study
Source: J Anim Ecol. 2016 Aug 31;85(6):1516–27. doi: 10.1111/1365-2656.12580 (PMC5298041; doi:10.1111/1365-2656.12580)
Supplement: Supplementary file 1 — Appendix S1. Supplementary Methods. Fig. S1. Example of the classification in six states of a 24 h‐period during the breeding season. Fig. S2. (a) Example of behavioural classification of saltwater‐immersion data by a HMM for a day in the wintering period of a bird. Table S1. Number of complete tracks collected during each period of the annual cycle (excluding pre‐laying and incubation), for experimental and non‐experimental years. Table S2. Comparison of activity budgets during the wintering period. [Correction added after online publication on 19 September 2016: Supporting information file replaced, as original was corrupt]. [file JANE-85-1516-s001.pdf]

ELECTRONIC SUPPLEMENTARY MATERIAL

Supplementary Methods

Filters applied on position data

Light data were decompressed and processed using the BASTrack software suite (British Antarctic Survey) and MatLab R2010b (MathWorks Inc.). Daily average positions were calculated, and we filtered out those with high standard error (longitude > 40 km, latitude > 30 km), and applied a speed filter of 1000 km per day (18h of sustained flight at average speed 55 km/h, Guilford *et al.* 2009).

Identification of at-sea behaviour from saltwater-immersion data

We used similar methods to (Dean *et al.* 2012) to identify behaviours from saltwater-immersion data during the wintering period and the breeding season with Hidden Markov Models (HMMS). For the wintering period HMMs were run on (normalised, logit-transformed) activity data and (arcsin-transformed) proportion of each day spent dry. For the breeding season, light data collected by geolocators was added as an extra variable, as it can help identify presence at the colony during the day (dark period during daylight hours). The optimal number of states for each period was first determined by running models on control data with 1-10 states. We initialised all models using uniform prior and transition probability matrices (i.e. all states equally probable). The optimal number of states was chosen as the best compromise between minimising the log-likelihood of the model, and identifying behaviours which were sufficiently different to make biological sense. We found 3 optimum states for the wintering period, and 6 for the breeding season (4 in daytime, 2 in night-time, Figure S0). Other studies of similar datasets analysed with HMMs and other machine-learning techniques and validated by other types of data also found three states during the non-breeding season (Guilford *et al.* 2009; Dean *et al.* 2012; Freeman *et al.* 2013), which identified sitting on the water surface (mostly wet) and sustained flight (mostly dry). The intermediate state (short bouts of dry and wet states) was called “foraging”. Although it may also contain other types of behaviours and should be interpreted cautiously, this state is likely to include most foraging activity, as showed by a study using a similar classification combined with diving data which found that >95% of diving occurred in this state during the breeding season (Dean *et al.* 2012). During the breeding season we allocated to

each of the 6 states the following behaviours using the mean immersion time of the states, and observing temporal patterns: sitting on the water, sustained flight, foraging type 1 (short wet bouts in-between longer dry bouts, perhaps more associated with searching) and foraging type 2 (short dry bouts in-between longer wet bouts) during the day; sitting on the water (mostly wet) and visiting the colony (mostly dry) at night (Figure S1). Models were first trained with data from control birds and then applied to data from treatment birds and, when available, from non-experimental years. The most likely sequence of hidden states was calculated and each data point (corresponding to a 10-minute period) was allocated a state.

Unlike (Dean *et al.* 2012; Freeman *et al.* 2013) we did not have any other tracking data to validate our classification, therefore we also classified our data in 3 states with simple thresholds (sitting: <3% dry; flight: >97% dry; “foraging”: anything in-between), similar methods were used in a number of species (Yamamoto *et al.* 2008; Lecomte *et al.* 2010; Catry *et al.* 2011; Ramirez *et al.* 2013) including Manx shearwaters (Shoji *et al.* 2015). All results were comparable, therefore we only present below the results using the HMM classification.

Natural differences in lay date prior to the experiment and individual consistency in phenotypic quality

In the year of manipulation, natural differences in laying date existed between groups. On average during the year of manipulation, control birds laid 3.3 ± 0.7 days before the colony median lay date, “shorter effort” birds laid 7.9 ± 1.7 days after the colony median, and “longer effort” birds laid 6.4 ± 1.4 days before the colony median. The laying dates in the manipulation year ranged from 14 days before to 20 days after the colony median, close to the actual range of laying dates at this colony. Birds in the “lower effort” treatment group laid later than control birds (LMM: n_{control}=12, $n_{\text{lower effort}}$=12; parameter estimate = 11.3 ± 1.9, R^2 = 0.63, χ_1^2 = 23.4, P < 0.001) while birds in the “higher effort” treatment group laid earlier (despite a lack of significance the effect size is relatively important) (LMM: $n_{\text{higher effort}}$=14; parameter estimate = -3.2 ± 1.6, R^2 = 0.19, χ_1^2 = 3.5, P = 0.060).

Attributing the shifts in lay date and egg mass we observed in the “higher effort” pairs following manipulation to an effect of the manipulation requires the assumption that individuals are consistent in their quality and hence in their breeding variables such as laying date and egg mass (otherwise one could argue that the early laying birds chosen for the “higher effort” group were having a good year and simply returned to their average state the

next year, regardless of the manipulation). Individual consistency in laying date and egg mass has been shown by previous studies of Manx shearwaters (Brooke 1978, Brooke 1990), but we did our own analyses to check whether this was the case in three different ways.

First, the pattern in laying date observed in the experimental year was similar the previous year: “lower effort” birds laid significantly later than controls (LMM: $n_{control}$=9, $n_{lower\ effort}$=9; parameter estimate = 5.9 ± 2.4, R^2 = 0.36, χ_1^2 = 5.5, *P* = 0.019) while there was no significant difference (but an effect size similar to the experimental year) between control and “higher effort” birds (LMM: $n_{higher\ effort}$=9; parameter estimate = 1.3 ± 2.5, R^2 = 0.19, χ_1^2 = 0.3, *P* = 0.579), which indicate a lack of major changes in lay date between the experimental year and previous years.

Second, we tried to match pairs from the “higher effort” group to un-manipulated pairs which laid at the same time during the experimental year, to test whether the shift in lay date and egg mass observed in our study after manipulation was not due to these early laying birds returning to their normal average state. We could only match 5 “higher effort” pairs to un-manipulated pairs. The 5 “higher effort” pairs laid 7.8 ± 2.0 days later in the year after manipulation than their matched un-manipulated pairs (paired T-test, t= -3.30, df = 4, *P* = 0.029). Similarly, while the 5 “longer effort” pairs laid eggs 1.4 ± 1.0 g lighter than they did prior to manipulation, the un-manipulated pairs laid eggs 0.4 ± 1.0 g heavier, but the difference was not statistically significant (paired T-test, t= 1.09, df = 4, *P* = 0.306). While the sample sizes are small, these results suggest that the delay in lay date and decrease in egg mass we observed after manipulation in the “longer effort” pairs was indeed due to the manipulation, and not because the birds were in an “abnormal” state in the experimental year and returned to their normal state after a good year.

Finally, we used 44 un-manipulated pairs for which we had data on timing of laying and egg mass for 2 breeding seasons during the 2011-2014 period (this period covers all the data included in our study). On average, the difference in laying date between two years was 0.16 ± 0.81 days, not statistically significant (paired T-test, t=-0.2, df = 43, *P* = 0.845). The difference in egg mass was 0.23 ± 0.49 g, also not statistically significant (paired T-test, t=-0.5, df = 36, *P* = 0.645). These results held when we looked at pairs for which we knew lay date for 2 consecutive years and breeding success for at least the first of these 2 years (n=29). Successful breeders (n = 22) laid eggs of similar mass at a similar date the following year (lay date: 1.11 ± 1.13

days later, paired T-test, t=-1.0, df = 21, P = 0.319; egg mass: 0.17 ± 0.69 g heavier, paired T-test, t=-0.2, df = 14, P = 0.814). Unsuccessful breeders (n = 7) did the same (lay date: 0.86 ± 2.15 days later, paired T-test, t=-0.4, df = 6, P = 0.704; egg mass: 0.14 ± 1.31 g lighter, paired T-test, t=-0.1, df = 6, P = 0.914). These results support previous findings of individual phenotypic consistency in shearwaters.

Taken together, these results agree with previous studies and show that un-manipulated Manx shearwaters are consistent year to year in their laying date and egg mass. Therefore, we are confident that the results we observed in our study are due to the effect of the manipulation. Chick mass and fledging success are known to be strongly associated with laying date and egg size in shearwater species (Perrins 1966; Perrins, Harris & Britton 1973; Ramos *et al.* 2003), so the consistency in laying date and egg mass is likely to lead to consistency in the other variables.

Sample sizes

For several reasons sample sizes vary between analyses. To avoid confusion, here are some explanations about why numbers vary so much. Please note that all sample sizes are given for every analysis, in the main text or in Table 1.

For laying dates and breeding variables in general, all nests were rigorously monitored from hatching to fledgling, in the 2 experimental years (i.e. 62 nests in total). While many of the nests were monitored from the start of the season and also in previous and following years, some nests were only monitored from the incubation stage, i.e. we did not know the laying date. It would have been possible to make an approximation by substracting 51 days to hatching date (the average duration of incubation in this species), but we preferred only to include dates we knew with certainty. Sample sizes in the years before and after manipulation also tend to be smaller, because some birds did not return, or changed burrow and were not detected.

As far as the sample sizes during the winter are concerned, the decreasing sample sizes as winter passes are due to more and more devices failing to record data. As a result, we have a large number of autumn migration tracks (all devices were working at the end of the breeding season when we deployed them), but we lost a few devices during the wintering period, and a few more during the spring migration. Similarly, an unfortunate rate of failure

of devices in the years prior to the experiment led to smaller sample sizes for the within-group longitudinal comparisons in wintering at-sea behaviour.

References

Catry, P., Granadeiro, J.P., Ramos, J., Phillips, R.A. & Oliveira, P. (2011) Either taking it easy or feeling too tired: old Cory's Shearwaters display reduced activity levels while at sea. *Journal of Ornithology*, **152**, 549–555.

Dean, B., Freeman, R., Kirk, H., Leonard, K., Phillips, R.A., Perrins, C. & Guilford, T. (2012) Behavioural mapping of a pelagic seabird: combining multiple sensors and a hidden Markov model reveals the distribution of at-sea behaviour. *Journal of The Royal Society Interface*, **10**, 1–12.

Freeman, R., Dean, B., Kirk, H., Leonard, K., Phillips, R.A., Perrins, C. & Guilford, T. (2013) Predictive ethoinformatics reveals the complex migratory behaviour of a pelagic seabird, the Manx Shearwater. *Journal of The Royal Society Interface*, **10**, 1–8.

Guilford, T., Meade, J., Willis, J., Phillips, R.A., Boyle, D., Roberts, S., Collett, M., Freeman, R. & Perrins, C.M. (2009) Migration and stopover in a small pelagic seabird, the Manx shearwater *Puffinus puffinus*: insights from machine learning. *Proceedings - Royal Society.Biological sciences*, **276**, 1215–1223.

Lecomte, V.J., Sorci, G., Cornet, S., Jaeger, A., Faivre, B., Arnoux, E., Gaillard, M., Trouve, C., Besson, D., Chastel, O. & Weimerskirch, H. (2010) Patterns of aging in the long-lived wandering albatross. *Proceedings of the National Academy of Sciences of the United States of America*, **107**, 6370–6375.

Perrins, C.M. (1966) Survival of Young Manx Shearwaters Puffinus Puffinus in Relation to Their Presumed Date of Hatching. *Ibis*, **108**, 132–135.

Perrins, C.M., Harris, M.P. & Britton, C.K. (1973) Survival of Manx shearwaters Puffinus puffinus. *Ibis*, **115**, 535–548.

Ramos, J.A., Moniz, Z., Solá, E. & Monteiro, L.R. (2003) Reproductive measures and chick provisioning of Cory's Shearwater Calonectris diomedea borealis in the Azores. *Bird Study*, **50**, 47–54.

Ramirez, I., Paiva, V.H., Menezes, D., Silva, I., Phillips, R.A., Ramos, J.A. & Garthe, S. (2013) Year-round distribution and habitat preferences of the Bugio petrel. *Marine Ecology Progress Series*, **476**, 269–284.

Shoji, A., Aris-Brosou, S., Culina, A., Fayet, A.L., Kirk, H., Padget, O., Juarez_Martinez, I., Boyle, D., Nakata, T., Perrins, C.M. & Guilford, T. (2015) Breeding phenology and winter activity predict subsequent breeding success in a trans-global migratory seabird. *Biology Letters*, **11**, 20150671.

Yamamoto, T., Takahashi, A., Yoda, K., Katsumata, N., Watanabe, S., Sato, K. & Trathan, P.N. (2008) The lunar cycle affects at-sea behaviour in a pelagic seabird, the streaked shearwater, *Calonectris leucomelas*. *Animal Behaviour*, **76**, 1647–1652.

Supplementary Figures

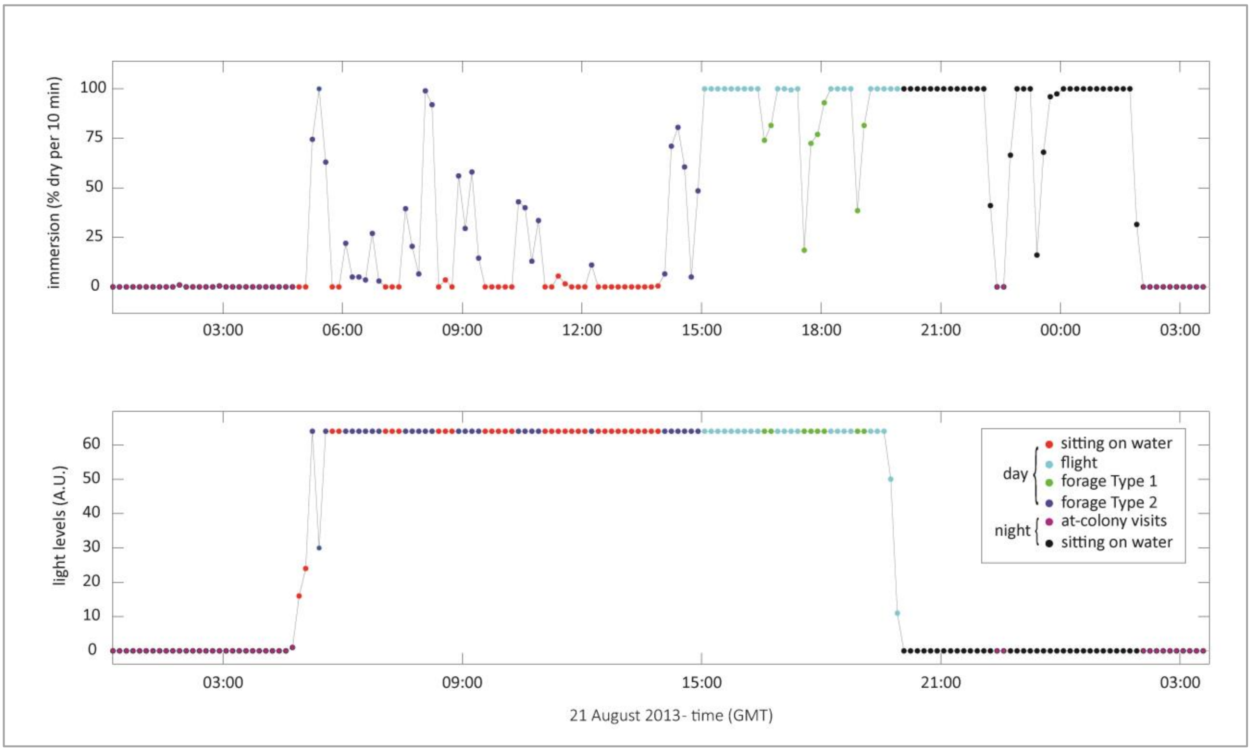

Figure S1. Example of the classification in 6 states of a 24h-period during the breeding season.

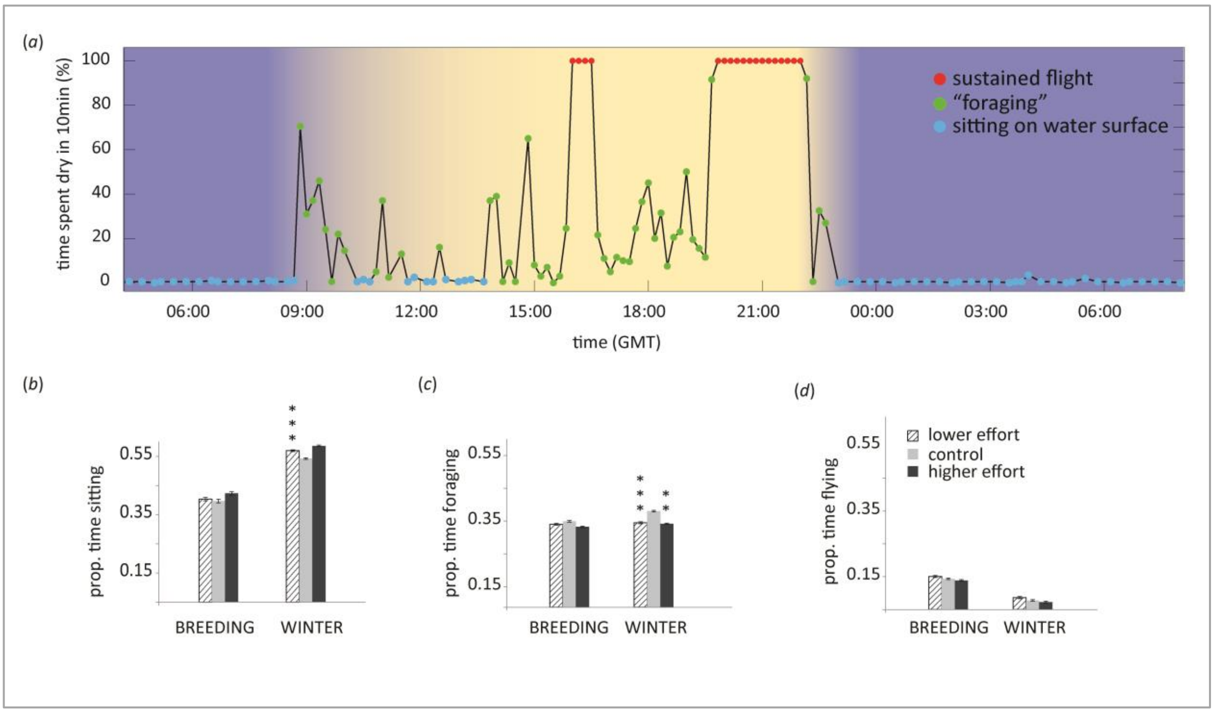

Figure S2. (*a*) Example of behavioural classification of saltwater-immersion data by a HMM for a day in the wintering period of a bird. Each point represents a 10min period. Points of low "dryness" are classified as one state (identified as sitting on the water surface, blue), while points of very high dryness are classified in a separate state (identified as sustained flight, red). Points of intermediate levels are classified in a 3rd intermediate state, which we called "foraging" but may also encompass other behaviours other than sustained sitting/flying. (*b -d*) Average proportions of time spent in the three behavioural states as classified by HMMs ((*b*) sitting; (*c*) foraging and (*d*) flying), for the breeding season and the overwintering period, for each group ("lower effort" treatment in white and black stripy pattern, control in light grey and "higher effort" treatment in dark grey). Difference with the Asterisks indicate significant differences between a treatment and the control group (*: $P < 0.05$; **: $P < 0.01$; ***: $P < 0.001$). Means ± SE.

Supplementary Tables

Table S1. Number of complete tracks collected during each period of the annual cycle (excluding pre-laying and incubation), for experimental and non-experimental years. Numbers within brackets represent the number of incomplete tracks (due to device failure).

* non-experimental years represent the 2011-2012 and 2013-2014 non-breeding seasons, with 12 (1) and 14 (0) tracks respectively.

	**Experimental years**			**on-experimental years (total) ***
	**2012 - 2013**	**2013 - 2014**	**Total**	
Autumn migration	40 (1)	46 (3)	86 (4)	26 (1)
Wintering	35 (5)	43 (3)	78 (8)	26
Spring migration	34 (1)	36 (7)	70 (8)	23 (3)
Chick-rearing	36	49	85	n/a

Table S2. Comparison of activity budgets during the wintering period. Comparisons are made between treatment and control groups in the winter after cross-fostering, and within-groups between experimental and non-experimental years (year immediately before and/or after). χ^2 and P values come from LMMs. The sample sizes are given under each treatment group. * Values during non-experimental years.

	**SITTING**		**FORAGING**		**FLYING**	
	mean ± SE (%)	within-year difference	mean ± SE (%)	within-year difference	mean ± SE (%)	within-year difference
**Comparisons with controls (within year)**						
Lower effort<br>$n = 20$	54.5 ± 3.7	$\chi_1^2 = 20.6$<br>$P < 0.001$	33.0 ± 3.2	$\chi_1^2 = 37.7$<br>$P < 0.001$	8.3 ± 1.9	$\chi_1^2 = 0.1$<br>$P = 0.924$
Control<br>$n = 26$	54.2 ± 2.8	n/a	38.0 ± 2.5	n/a	7.8 ± 1.4	n/a
Higher effort<br>$n = 30$	58.1 ± 2.6	$\chi_1^2 = 1.8$<br>$P = 0.17$	33.9 ± 2.0	$\chi_1^2 = 7.13$<br>$P = 0.008$	7.2 ± 1.2	$\chi_1^2 = 0.5$<br>$P = 0.477$
**Within-group comparisons (between years)**						
Lower effort<br>$n = 20$	54.5 ± 0.3<br>58.0 ± 0.5 *	$\chi_1^2 = 0.3$<br>$P = 0.609$	33.0 ± 0.3<br>34.6 ± 0.3 *	$\chi_1^2 = 0.5$<br>$P = 0.461$	8.3 ± 0.2<br>7.4 ± 0.2 *	$\chi_1^2 = 0.1$<br>$P = 0.767$
Control<br>$n = 26$	54.2 ± 0.2<br>53.9 ± 0.6 *	$\chi_1^2 = 5.1$<br>$P = 0.023$	38.0 ± 0.2<br>38.3 ± 0.5 *	$\chi_1^2 = 5.5$<br>$P = 0.019$	7.8 ± 0.3<br>7.8 ± 0.1 *	$\chi_1^2 = 0.1$<br>$P = 0.803$
Higher effort<br>$n = 30$	58.1 ± 0.2<br>59.3 ± 0.5 *	$\chi_1^2 = 4.7$<br>$P = 0.029$	33.9 ± 0.2<br>34.6 ± 0.4 *	$\chi_1^2 = 4.2$<br>$P = 0.041$	7.2 ± 0.1<br>6.1 ± 1.9 *	$\chi_1^2 = 0.5$<br>$P = 0.494$
